# Rare Cause of Acute Loss of Vision in a Patient With Sickle Cell Trait

**DOI:** 10.7759/cureus.42535

**Published:** 2023-07-27

**Authors:** Srikaran Bojja, Nismat Javed, Nishant Allena, Shreya Bojja, Misbahuddin Khaja

**Affiliations:** 1 Internal Medicine, BronxCare Health System, Icahn School of Medicine at Mount Sinai, New York City, USA; 2 Pulmonology, BronxCare Health System, Icahn School of Medicine at Mount Sinai, New York City, USA; 3 Medicine, Mallareddy Institute Of Medical Sciences, Hyderabad , IND; 4 Internal Medicine/Pulmonary Critical Care, BronxCare Health System, Icahn School of Medicine at Mount Sinai, New York City, USA

**Keywords:** hydroxyurea, goldberg's classification, vitrectomy, sickle cell trait, red cell exchange transfusion, exchange transfusion, sickle cell retinopathy, vitreous hemorrhage

## Abstract

Sickle cell disease (SCD) is a prevalent inherited blood disorder with various ocular manifestations, including sickle cell retinopathy (SCR), characterized by retinal microcirculation impairment and ischemic complications. We present the case of a 21-year-old male with sickle cell trait who experienced a sudden, painless loss of vision in his left eye. Ophthalmologic examination revealed vitreous hemorrhage and neovascularization, indicating SCR. Initial treatment with hydroxyurea and exchange transfusions led to partial improvement. However, due to persistent vitreous hemorrhage, the patient underwent a vitrectomy. The sickle cell trait affects a large global population, and its retinopathy is a rare but severe complication. The pathogenesis and risk factors for SCR are similar to those for SCD. The diagnosis of SCR is established through fundoscopic examination and graded based on Goldberg's classification. Management involves a multidisciplinary approach targeting systemic illness and visual defects, including hydroxyurea, photocoagulation, anti-vascular endothelial growth factors, and vitrectomy. Awareness, early diagnosis, and timely intervention are essential to preventing vision-threatening complications in sickle cell trait patients with SCR.

## Introduction

Sickle cell disease (SCD) is the most prevalent inherited blood disorder, characterized by ongoing hemolysis, damage to blood vessels, and reduced blood flow to various organs, leading to complications in multiple systems within the body [1]. In Black individuals in North America, the SCD incidence is close to 0.5%, and about 7-8% of the same population are carriers of the sickle cell trait [2]. Among the various ocular manifestations of SCD, sickle cell retinopathy (SCR) stands out as one of the most severe. This occurs due to the progressive impairment of the retina's microcirculation, resulting in ischemic maculopathy and occlusions in peripheral areas [3, 4]. Sickle cell retinopathy is commonly seen in adults rather than children [5]. The progression of SCR can be divided into five stages, varying from non-proliferative types to proliferative types [6]. Sickle cell retinopathy may cause a wide spectrum of ophthalmologic findings on examination, which include characteristic retinal pigmentary changes or iridescent spots, "salmon patch" hemorrhages in the periphery of the retina, sunbursts, retinoschisis cavities, vessel tortuosity, angioid streaks, ischemic changes in the choroid, optic disc-comma signs, macular vascular occlusions, central retinal artery or vein occlusions, or any of the signs of proliferative SCR [4].

Here, we present the case of a 21-year-old male with a medical history of sickle cell trait who presented with acute painless loss of vision and was found to have a vitreous hemorrhage, which further required complex management.

## Case presentation

**Figure 1:**
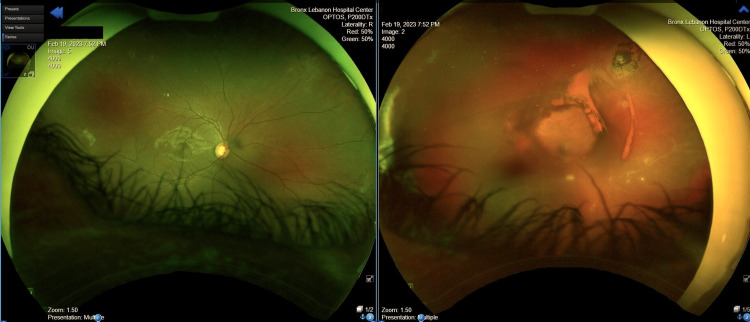
Initial fundoscopic examination reveals left vitreous hemorrhage

A 21-year-old male with a medical history of sickle cell trait presented to the emergency department with a six-hour history of painless loss of vision in the left eye. He reported seeing moving clouds and blurry vision prior to the episode. He reported non-adherence to his medications, including hydroxyurea 1500 mg, for the past three years. He denied any history of smoking or drug abuse. Family history was significant for hypertension, glaucoma, and sickle cell trait. Vitals revealed a temperature of 98.8 °F, a pulse of 96 beats per minute, a blood pressure of 134/66 mmHg, and an oxygen saturation of 98% in room air. A physical exam revealed the absence of pupillary light response and accommodation in the left eye, mild bilateral scleral injection, and significant visual loss in the left eye. Ophthalmology was consulted, and evaluation revealed a large vitreous hemorrhage obscuring the nerve and macula (Figure 1) and neovascularization of supratemporal and infratemporal regions in the left eye.

Neovascularization changes were also noted in the right eye; fundoscopic imaging findings are tabulated in Table 1, suggesting vitreous hemorrhage due to stage 4 sickle cell retinopathy (SCR) in the left eye and stage 3 SCR in the right eye.

**Table 1:** Initial fundoscopic findings OD: oculus dexter; OS: oculus sinister

	OD	OS
Disc	(-) hemes, normal color/contour	No view; only a blurred image of the disc is visible
Choroid	Normal	Not visualized
Retina	Flat	Flat retina; poor view
Macula	Flat	No view
Vessels	Neovascularization, Ischemic perpherally	Neovascularization visible infratemporally, scar/fibrovascular neovascularization supratemporally
Peripheral	A small region of sea-fanning neovascularization (with white vessels and fibrovascular appearance) supranasally, second region of neovascularization infratemporally	Sea-fanning neovascularization visible infratemporally, scar/fibrovascular neovascularization supratemporally
Vitreous	Clear	Vitreous hemorrhage

Initial investigations in Table 2 revealed thrombocytosis, elevated albumin levels, and hyperbilirubinemia, predominantly indirect.

**Table 2:** Initial lab investigations

Variable	Value	Reference value
Hemoglobin	14.8 g/dl	12.0-16.0 g/dl
Hematocrit	42.6 %	42.0-51.0 %
Mean corpuscular volume	88.2 fL	80.0-96.0 fL
White blood cell	7.6 k/ul	4-8-10.8 k/ul
Platelet count	433 k/ul	150-400 k/ul
Reticulocyte %	3.1%	0.5-1.5%
Prothrombin time	13.4 seconds	9.9-13.3 seconds
Partial thromboplastin time	33.3 seconds	27.2-39.6 seconds
International normalized ratio	1.12	0.85-1.14
Sodium	138 mEq/L	135-145 mEq/L
Potassium	4.2 mEq/L	3.5-5.0 mEq/L
Chloride	101 mEq/L	98-108 mEq/L
Blood urea nitrogen	6.0 mg/dL	8.0-26.0 mg/dL
Creatinine	0.8 mg/dL	0.5-1.5 mg/dL
Calcium	10.0 mg/dL	8.5-10.5mg/dL
Albumin	5.2 g/dL	3.4-4.8 g/dL
Total bilirubin	2.7 mg/dL	0.2-1.2 mg/dL
Direct bilirubin	0.4 mg/dL	0.0-0.3 mg/dL
Alanine aminotransferase	10 unit/L	5-40 unit/L
Aspartate transaminase	17 unit/L	9-48 unit/L
Alkaline phosphatase	92 unit/L	53-128 unit/L
Total protein	7.9 g/dL	6.0-8.5 g/dL
Serum iron	71 ug/dL	65-175 ug/dL
Unsaturated iron binding capacity	228 ug/dL	112-346 ug/dL
Serum ferritin	597 ng/mL	13.0-150 ng/mL

A CT angiography of the brain did not reveal any significant abnormalities. Hematology was consulted and recommended restarting hydroxyurea at 1500 mg once daily and initiating exchange transfusion considering the diagnosis of vitreous hemorrhage and extensive bilateral sickle cell retinopathy. The patient was admitted to the intensive care unit (ICU) for monitoring.

The patient received four cycles of exchange transfusions with packed red blood cells. Significant clinical improvement was noted after the therapy. He was discharged with outpatient hematology and ophthalmology follow-up. On outpatient ophthalmology evaluation, improvement was visualized in the size of the vitreous hemorrhage; however, it did not completely resolve after one month. Therefore, the patient underwent left-sided pars plana vitrectomy (PPV) and endolaser and endodiathermy, has been in close follow-up with the ophthalmology clinic, and has experienced an improvement in vision.

## Discussion

The sickle cell trait affects a large proportion of people globally. Estimates from recent studies reveal that 300 million people worldwide have sickle cell trait [7]. In the US, prevalence rates are about 9% for African Americans and 0.2% for Caucasians [8]. The prevalence is higher in certain geographical territories, specifically areas where malaria is endemic in tropical and sub-tropical regions [9]. Estimates for sickle cell retinopathy reveal that it impacts about 90,000-100,000 Americans every year [10]. However, most cases are noted with sickle cell disease, and the phenomenon is very rare in patients with sickle cell trait [11].

The pathogenesis of retinopathy in sickle cell trait has a similar postulated mechanism as for sickle cell disease. The cascade of hypoxia, acidosis, and ischemia promotes angiogenesis and can subsequently result in morbidity and mortality in this cohort [11]. Risk factors, including smoking, older age at presentation, high hemoglobin levels, and leukocytosis, were associated with progressive and non-progressive sickle cell retinopathy [4]. Our patient, however, did not have any such risk factors.

Symptoms suggestive of retinopathy include abrupt vision loss or visualization of floaters [10,11]. In most cases, patients had a concomitant uncontrolled systemic disease that increased the progression of the disease [11, 12-14]. However, our case did not have any such systemic illnesses. The diagnosis was usually confirmed by fundoscopic examination [11,12-14]. 

Variable findings have been documented on examination [11,12-14] that are graded based on Goldberg’s classification [6]. Stage I, defined as peripheral arterial occlusion, is caused by the aggregation of defective hemoglobin within erythrocytes [6]. Stage II, defined as peripheral arteriovenous anastomoses, is suggestive of circulatory disturbances [6]. Stages III, IV, and V are defined as sea-fan neovascularization, vitreous hemorrhage, and tractional retinal detachment, respectively [6]. In this case, the patient showed signs of both stage III and IV sickle cell retinopathy. Additional ophthalmological investigations, including B-scan, optical coherence tomography, and fluorescein angiography, can help in better visualization of the same lesions [11].

Management requires a multidisciplinary approach to target both systemic illnesses and visual defects. Hydroxyurea promotes the production of hemoglobin F, diluting the percentage of deformed red blood cells and decreasing further risk for complications [15,16]. In cases of neovascularization, scatter photocoagulation can be considered if the areas do not undergo auto-infarction [15]. However, the techniques are associated with an increased risk of vitreous hemorrhage [15]. Intravitreal injection of anti-vascular endothelial growth factors is another possible treatment option. Although bevacizumab has been discussed in this regard with patients undergoing regression of neovascularization, secondary hyphema was reported as a possible complication [15]. Lastly, in refractory cases of neovascularization resulting in vitreous hemorrhage, vitrectomy has proven to be of immense importance [15].

## Conclusions

In conclusion, this case report highlights the severity and complexity of SCR in a patient with sickle cell trait. Sickle cell retinopathy, although rare in individuals with sickle cell trait, can lead to vision-threatening complications, emphasizing the need for increased awareness and early diagnosis in this population. Effective management of SCR requires a multidisciplinary approach. Timely diagnosis and appropriate interventions are essential to preventing irreversible visual impairments in affected individuals. Furthermore, future research should focus on understanding the underlying mechanisms of SCR in sickle cell traits and exploring novel therapeutic approaches to optimize patient outcomes.

## References

[1] Moussa O, Chen RW (2021). Hemoglobinopathies: ocular manifestations in children and adolescents. Ther Adv Ophthalmol.

[2] McCavit TL (2012). Sickle cell disease. Pediatr Rev.

[3] Elagouz M, Jyothi S, Gupta B, Sivaprasad S (2010). Sickle cell disease and the eye: old and new concepts. Surv Ophthalmol.

[4] Nawaiseh M, Roto A, Nawaiseh Y (2022). Risk factors associated with sickle cell retinopathy: findings from the Cooperative Study of Sickle Cell Disease. Int J Retina Vitreous.

[5] Bonanomi MT, Lavezzo MM (2013). Sickle cell retinopathy: diagnosis and treatment. Arq Bras Oftalmol.

[6] Goldberg MF (1971). Classification and pathogenesis of proliferative sickle retinopathy. Am J Ophthalmol.

[7] Gibson JS, Rees DC (2016). How benign is sickle cell trait?. EBioMedicine.

[8] El Ariss AB, Younes M, Matar J, Berjaoui Z (2016). Prevalence of sickle cell trait in the southern suburb of Beirut, Lebanon. Mediterr J Hematol Infect Dis.

[9] Ashorobi D, Ramsey A, Yarrarapu SNS, Bhatt R (2023). Sickle Cell Trait. https://www.ncbi.nlm.nih.gov/books/NBK537130/..

[10] Parekh PK, Miller MA, Russell SR (2016). Sickle cell retinopathy. EyeRounds.org.

[11] Reynolds SA, Besada E, Winter-Corella C (2007). Retinopathy in patients with sickle cell trait. Optometry.

[12] Vangipuarm G, Saraf SS, Zhang Q, Wang R, Rezaei KA (2020). Profound presentation of retinopathy in a patient with sickle cell trait and diabetes mellitus. J Ophthalmic Vis Res.

[13] Caranfa JT, Witkin AJ (2023). Severe proliferative retinopathy in a patient with sickle cell trait. Am J Ophthalmol Case Rep.

[14] Ghods S, Pour EK, Faghihi H (2021). Sickle cell trait presenting as unilateral proliferative retinopathy and macular thinning in a pregnant woman. Case Rep Ophthalmol Med.

[15] Abdalla Elsayed ME, Mura M, Al Dhibi H, Schellini S, Malik R, Kozak I, Schatz P (2019). Sickle cell retinopathy. A focused review. Graefes Arch Clin Exp Ophthalmol.

[16] Sheikh AB, Nasrullah A, Lopez ED (2021). Sickle cell disease-induced pulmonary hypertension: a review of pathophysiology, management, and current literature. Pulse (Basel).

